# Clinical anxiety among a sample of dental students in South Sinai

**DOI:** 10.1186/s12903-025-05532-2

**Published:** 2025-02-08

**Authors:** Christine Raouf George Mikhail, Mai Hamdy Ragab, Yousra Ahmed, Eman D. El Desouky, Fatma E. A. Hassanein, Mohamed Bekhit, Marwa Hassan Mostafa

**Affiliations:** 1 Department of Oral Medicine, Diagnosis and Periodontology, Faculty of Dentistry, Fayoum University, Fayoum, Egypt; 2 Department of Oral Medicine, Diagnosis and Periodontology, Faculty of Dentistry, King Salman International University, EL Tor, South Sinai Egypt; 3 Faculty of dentistry, Suez canal university, El Sheikh Zayed, Egypt; 4 Department of Removable Prosthodontics, Faculty of Dentistry, King Salman International University, EL Tor, South Sinai Egypt; 5 Epidemiology and Biostatistics, NCI, Cairo University, Cairo, Egypt; 6 Department of Oral and Maxillofacial surgery, Faculty of Dentistry, King Salman International University, EL Tor, South Sinai Egypt; 7 Department of Fixed and Removable Prosthodontics, Oral And Dental Research Institute, National Research Centre, Cairo, Egypt

**Keywords:** Clinical anxiety, Stress, Medical students, Dental students, Dental procedures, Clinical situations

## Abstract

**Background:**

Anxiety may be induced in the dental clinics, which is an essential learning environment for undergraduate dental students. This could have a negative impact on clinical performance. This study aimed to estimate the incidence of clinical anxiety among a sample of Dental students and to determine possible precipitating factors for clinical anxiety among them.

**Methods:**

A cross-sectional study was carried out among 3rd and 4th year dental students at King Salman International University using the modified 34 items of Moss and McManus clinical anxiety questionnaire.

**Results:**

263 students participated. Dental students reported higher incidence of clinical anxiety (60.8%), particularly with different dental treatment plan procedures related clinical situations fulfilling 31.2 out of mean score of anxiety all (74.0 ). Females had higher anxiety score in all domains than males, the same trend noticed in students who reported dental phobia and previous bad dental experiences (*P* < 0.001, *P* 0.016, *P* 0.003 respectively). Participants rated the clinical anxiety precipitating situations in a descending order as follows: extracting wrong tooth (39.5%), giving wrong treatment (37.6%), tearing of the cheek/lips due to catching on a dental burr (35.7%), fracturing a tooth (34.4%), wrong diagnosis (28.1%), inadvertently hurting the patient (22.1%), inability to meet requirements before exams (19.1%), dealing with a fainting patient during dental treatment (18.7%). Two clinical anxiety precipitating situations had almost the same score including restoration failure/recurrent caries and endodontic retreatment procedures 14.8% and 14.4% respectively. Also, extracting tooth and arresting postoperative bleeding had the same score of 13.7%, while the rest of other clinical situations ranged from 11.8 to 1.1%.

**Conclusions:**

dental students had a comparatively high level of anxiety during clinical classes especially females and those with dental phobia and previous bad dental experiences. Dental students may benefit from stress management classes, mentorship programs, and improved clinical supervision in order to reduce clinical anxiety and build resilience.

## Background

Anxiety permeates dental students’ experiences and is typified by tension, concern, and physiological changes including elevated blood pressure. It’s critical to separate anxiety from dread because the former is more focused on the future and encompasses a wide range of dangers, anxiety is triggered instantly and is linked to particular dangers [1]. People who struggle with anxiety disorders frequently deal with intrusive thoughts that don’t go away and may avoid particular situations out of fear. When it comes to healthcare training, prospective healthcare professionals experience a particular kind of anxiety during the shift from preclinical to clinical stages [2].

The first goal of medical school is to become an expert in theory; the second is to work practically with patients and carry out basic therapeutic operations. Even while lecturers provide professional supervision, undergraduate students should nonetheless perform several operations on their own [3, 4].

Dental students face many challenges when it comes to providing treatment under supervision, which differs greatly from treating phantom heads and meeting clinical standards. A conceptual framework of dental professionalism among dental students that includes the following eight areas was also described by SHAW et al. [5]: understands self; understands others; trustworthiness; ability to relate to context; vocational; altruistic; reliability; and account ability. The cumulative effect of prior experiences and occurrences shapes the public’s and patients’ perceptions of the dentistry profession. Regarding dental students, their views about dentistry can influence their academic performance and directly impact their practice. Additionally, dental students describe a variety of significant factors that influenced their decision to pursue dentistry, with differences by sex and wealth Ott [6].

Positive stress is brief, mild, and can return to normal within acceptable time, while toxic stress is intense, continuous, and irreversible, damaging neuropsychomotor development and increasing disease risk [7]. Clinical anxiety is expected given that dentistry education is widely viewed as challenging, with stresses originating from the environment, training, and relationships, very few studies have been conducted on dentistry students to identify the conditions that cause anxiety, in contrast to many studies on medical and nursing students [8].

In a study to identify the conceptions of dentists and dental students (DSs) about paediatric dental patients, Buldur defined a PDP as unpredictable, dangerous, uncontrollable, requiring care and sensitivity, valuable, and orientable. The most important factors leading to these conceptions were the uncooperativeness of some PDPs and the effectiveness of behavior management [9].

When Moss and McManus (1992) [10]examined the fears of first-year clinical medical students, they found that participants had moderate to severe anxiety in almost half of all clinical scenarios. From the pre-clinical to the clinical phases of their education, medical students’ anxiety development varied by gender, revealed by Greenfield et al. [11], wherein women reported a noticeably greater number of clinical scenarios that caused anxiety.

The University of Otago conducted a study among dental students which indicated that the general clinical scenarios that were linked to the highest levels of anxiety were making a mistake in a diagnosis, injuring patients, handling medical emergencies, and becoming infected. The possibility of undergoing surgery, particularly circumstances that contributed to increased anxiety in the students were temporomandibular joint issues and unsuccessful local analgesia [12].

The year of study was substantially correlated with the degree of anxiety expressed by administering an inferior alveolar nerve block and managing poor oral hygiene, with the lower class exhibiting higher levels of worry. The discovery that clinical anxiety may hinder dental students’ ability to receive high-quality instruction and perform well served as further evidence that It is necessary to assess clinical anxiety in dental students from the viewpoints of both students and instructors and to provide examples of strategies for reducing it [8].

Lack of knowledge from the instructors regarding the circumstances that dentistry students believed to cause anxiety. Additionally, previous studies didn’t allow for the assessment of differences and similarities or the formulation of a well-informed prescription aimed at reducing anxiety and enhancing training in general [2]. Therefore, the objectives of conducting such study were to estimate the incidence of clinical anxiety among a sample of Dental students from 3rd and 4th years and to determine possible precipitating factors/situations for clinical anxiety among them.

## Methods

Adhering to the STROBE statement checklist when documenting observational cross-sectional research.

An observational cross-sectional study was done at the faculty of Dentistry, King Salman International University, El Tor, South Sinai, Egypt. A straightforward consecutive sampling technique was used to recruit 263 dentistry students. Participants were chosen over the course of three months, from February to April 2024, and represented the third and fourth academic years of dental students at King Salman International University’s Faculty of Dentistry. The participants ranged in age from 19 to 24.

A systematic questionnaire, a modified version of the Moss and McManus clinical anxiety questionnaire [6], was used to evaluate each participant. It contained 34 items covering particular dental procedures thought to be causes of clinical anxiety. A 4-point Likert scale, ranging from “not anxious,” “slightly anxious,” “fairly anxious,” and “very anxious,” was used to evaluate the situations. Responses were ranked 1 for not anxious, 2 for slightly anxious, 3 for fairly anxious, and 4 for very anxious for analysis purposes. Additionally, demographic data like gender and age were gathered. Additionally, dental history was documented, including any dental phobias, prior negative dental experiences, and oral hygiene status.

The primary outcome was the estimation of the clinical anxiety’s incidence among a sample of dental students. The secondary outcome was the identification of the potential precipitating clinical situations for this anxiety among these students at the Faculty of Dentistry, King Salman International University, El Tor, South Sinai, Egypt.

### Sample size calculation and statistical methods

Based on earlier research by Fathima et al., Gazal et al., and Basudan et al. [2, 9, 10], the sample size is determined. 263 individuals were enrolled using a power of 80%, a significance threshold of 5%, a 90% confidence interval, and the total number of third and fourth grade pupils. Epi Info is used to determine the sample size. All the results were subjected to statistical analysis. Statistical analysis was done using Statistical Package for Social Sciences, Version 27.0 (SPSS, IBM) for Windows. Continuous variables were summarized as mean values ± standard deviation (SD) or median (range) as appropriate. Percentages were calculated for categorical data. We computed percentages for categorical data.

### Methods and annex

Questions were classified into three domains according to general and specific clinical anxiety precipitating situations(Table 1). Three Domains were examined as presented in (Table 2). Cronbach’s alpha coefficient was calculated to assess the internal consistency of the questionnaire items. Cronbach’s alpha ranges from 0 to 1, with higher values indicating greater internal consistency. Cutoff values for interpreting Cronbach’s alpha were based on the guidelines provided by George and Mallery (2003): ≥ 0.9 is considered “excellent”, 0.8–0.9 is “good”, 0.7 to 0.8 is “acceptable”, 0.6 to 0.7 is “questionable”, 0.5 to 0.6 is “poor”, and below 0.5 is “unacceptable”.

**Table 1:** Classification of questions into 3 main domains

1st	**General Clinical management requirements**
1	Presenting cases in clinical session
2	Inability to meet requirements before exams
3	Coping with unco-operative patient
4	Dealing with psychiatric patients
5	Dealing with geriatric patients
6	Dealing with a fainting patient during dental treatment
7	Patients follow up after any treatment as indicated
8	Inadvertently hurting the patient
2nd	**Patient’s Diagnosis and management**
1	Taking history from patient (chief complaint, medical and dental history)
2	Extra-oral examination of patient
3	Intra-oral examination of patient
4	Indicating required investigations
5	Taking radiographs
6	Interpreting radiographs
7	Interpreting blood investigations
8	Reaching a definitive diagnosis
9	Wrong diagnosis
10	Managing medically compromised patients
11	Referral of patients for medical consultation
12	Prescribing medication
13	Giving a wrong treatment
3rd	**Different dental treatment plan procedures**
1	Scaling procedures
2	Administering local anesthesia
3	Extracting tooth
4	Arresting postoperative bleeding
5	Fracturing a tooth
6	Extracting wrong tooth
7	Operative treatment procedures
8	Accidental pulp exposure
9	Tearing of the cheek/lips due to catching on a dental burr
10	Restoration failure/recurrent caries
11	Endodontic treatment procedures
12	Endodontic retreatment procedures
13	Removable and fixed prosthodontics procedures

**Table 2:** Examination of three domains

		No. of items	Min. score	Max. score	Cronbach’s alpha	Interpretation
1st	Clinical management requirements in general	8	8	40	0.81	Good
2nd	Patient’s Diagnosis and management	13	13	65	0.88	Good
3rd	Different dental treatment plan procedures	13	13	65	0.91	Excellent
	Overall	34	34	170	0.95	Excellent

## Results

Of the 263 participants, 178 (67.7%) were in the 3rd grade while 85 (32.3%) were in the 4th grade. The mean age of participants was 20.9 years with a standard deviation of 0.9 years, ranging from 19 to 24 years. Among the respondents, 62.7% were male and 37.3% were female. 87.1% of participants grew up in Egypt, while 12.9% grew up outside Egypt. 78.7% of participants didn’t have medical or dental professional family members. Among those with disorders, 66.7% had psychiatric disorders and 33.3% had medical disorders. 90.5% of participants didn’t take any medications. 68.4% of participants visited the dentist frequently, 31.6% didn’t visit at all, while others visited at different frequencies. 87% of participants had good oral hygiene, with smaller percentages falling into category like bad or poor. 88.2% of respondents didn’t suffer from dental phobia, and 87.5% didn’t have previous bad dental experiences.

The assessment of the responses to the modified version of Moss and McManus [10], including 34 questions to cover specific dental procedures perceived as sources of clinical anxiety was identified as follow (Table 3).

**Table 3:** Anxiety questionnaire

	Not Anxious	Slightly	Fairly	Very Anxious
	*n*(%)	*n*(%)	*n*(%)	*n*(%)
Presenting cases in clinical session	94(35.7)	114(43.3)	42(16)	13(4.9)
Inability to meet requirements before exams	42(16)	88(33.6)	82(31.3)	50(19.1)
Coping with unco-operative patient	64(24.3)	107(40.7)	73(27.8)	19(7.2)
Dealing with psychiatric patients	66(25.1)	109(41.4)	63(24)	25(9.5)
Dealing with geriatric patients	107(40.7)	89(33.8)	51(19.4)	16(6.1)
Taking history from patient (chief complaint, medical and dental history)	185(70.3)	57(21.7)	17(6.5)	4(1.5)
Extra-oral examination of patient	179(68.1)	66(25.1)	14(5.3)	4(1.5)
Intra-oral examination of patient	156(59.3)	86(32.7)	18(6.8)	3(1.1)
Indicating required investigations	143(54.4)	93(35.4)	22(8.4)	5(1.9)
Taking radiographs	139(52.9)	85(32.3)	32(12.2)	7(2.7)
Interpreting radiographs	108(41.1)	99(37.6)	45(17.1)	11(4.2)
Interpreting blood investigations	93(35.4)	95(36.1)	64(24.3)	11(4.2)
Reaching a definitive diag1sis	86(32.8)	99(37.8)	60(22.9)	17(6.5)
Managing medically compromised patients	58(22.1)	99(37.6)	76(28.9)	30(11.4)
Referral of patients for medical consultation	96(36.6)	95(36.3)	54(20.6)	17(6.5)
Prescribing medication	100(38)	95(36.1)	49(18.6)	19(7.2)
Scaling procedures	135(51.5)	86(32.8)	33(12.6)	8(3.1)
Administering local anesthesia	68(26)	93(35.5)	70(26.7)	31(11.8)
Extracting tooth	66(25.1)	87(33.1)	74(28.1)	36(13.7)
Arresting postoperative bleeding	53(20.2)	97(37)	76(29)	36(13.7)
Operative treatment procedures	92(35)	110(41.8)	41(15.6)	20(7.6)
Endodontic treatment procedures	64(24.4)	105(40.1)	66(25.2)	27(10.3)
Endodontic retreatment procedures	59(22.4)	88(33.5)	78(29.7)	38(14.4)
Removable and fixed prosthodontics procedures	96(36.5)	100(38)	39(14.8)	28(10.6)
Dealing with a fainting patient during dental treatment	57(21.8)	75(28.6)	81(30.9)	49(18.7)
Patients follow up after any treatment as indicated	113(43)	73(27.8)	46(17.5)	31(11.8)
Inadvertently hurting the patient	49(18.6)	72(27.4)	84(31.9)	58(22.1)
Wrong diagnosis	26(9.9)	70(26.6)	93(35.4)	74(28.1)
Giving a wrong treatment	24(9.1)	51(19.4)	89(33.8)	99(37.6)
Fracturing a tooth	25(9.5)	62(23.7)	85(32.4)	90(34.4)
Extracting wrong tooth	28(10.6)	38(14.4)	93(35.4)	104(39.5)
Accidental pulp exposure	26(9.9)	54(20.5)	103(39.2)	80(30.4)
Tearing of the cheek/lips due to catching on a dental burr	33(12.5)	55(20.9)	81(30.8)	94(35.7)
Restoration failure/recurrent caries	50(19)	106(40.3)	68(25.9)	39(14.8)

Concerning the incidence of clinical anxiety among the participants, 160 (60.8%) of the participated dental students were clinically anxious, while the rest were non anxious clinically. The mean score for anxiety all was 74.0 with the 3rd domain clinical situations related questions (i.e. different dental treatment plan procedures) fulfilling 31.2 out of 74.0 followed by the 2nd domain (Patient’s Diagnosis and management) representing 25.4, then the 1st domain (Clinical management requirements in general) 17.7 out of anxiety all (Table 4).

**Table 4:** Anxiety all score

	Mean	SD	Median	Minimum	Maximum
1st domain (Clinical management requirements general)	17.7	5.0	18.0	8.0	31.0
2nd domain (Patient’s Diagnosis and management)	25.4	7.1	24.0	13.0	50.0
3rd domain (Different dental treatment procedures)	31.2	8.6	31.0	13.0	50.0
Anxiety all	74.0	19.0	73.0	34.0	122.0

Females had higher anxiety score in all domains than males, the same trend noticed in students who reported dental phobia and previous bad dental experiences, which were statistically significant *P* < 0.001, *P* < 0.016, *P* < 0.003 respectively (Table 5).

**Table 5:** Anxiety in relation to demographic variables, dental phobia and previous bad experiences

		Not Anxious (≤ 50%)	Anxious (> 50%)	*P* value
		*n* = 130(%)	*n* = 160(%)	
Age (yrs.)	Mean ± SD	20.9 ± 0.9 19–24		0.123
	Range			
Gender	Female	24(24.5)	74(75.5)	**< 0.001**
	Male	79(47.9)	86(52.1)	
Family doctors	No	77(37.2)	130(62.8)	0.209
	Yes	26(46.4)	30(53.6)	
Disorder	No	87(41.6)	122(58.4)	0.107
	Yes	16(29.6)	38(70.4)	
Medical	No	98(40.0)	147(60.0)	0.305
	Yes	5(27.8)	13(72.2)	
Psychiatry	No	92(40.5)	135(59.5)	0.255
	Yes	11(30.6)	25(69.4)	
Medications	No	92(38.7)	146(61.3)	0.603
	Yes	11(44.0)	14(56.0)	
Do you frequently visit dentist	No	32(38.6)	51(61.4)	0.891
	Yes	71(39.4)	109(60.6)	
Oral hygiene	Bad- Not good	8(44.4)	10(55.6)	0.212
	Fair- Moderate	2(22.2)	7(77.8)	
	Good- Normal	86(37.9)	141(62.1)	
	Excellent	5(71.4)	2(28.6)	
Phobia	No	97(41.8)	135(58.2)	**0.016**
	Yes	6(19.4)	25(80.6)	
Any previous bad dental experiences	No	98(42.6)	132(57.4)	**0.003**
	Yes	5(15.2)	28(84.8)	

*P* < 0.05 is statistically significant, analysis done by independent t test

## Discussion

It is well known that dentistry causes a great deal of stress for dental students, especially those pursuing undergraduate degrees. Anxiety can be especially high during the curriculum’s shift from pre-clinical to clinical phases [13]. Several examples, including anxiety can be raised by exams, pre-clinical work, clinical performance reviews, learning the required skills, and caring for patients with special needs. Clinical anxiety can be triggered by seemingly straightforward duties like taking patients’ pulse rates or interacting with them. To boost students’ confidence and raise the calibre of knowledge they acquire, it is essential to identify any conditions that could cause anxiety throughout the learning process [14]. Prior research has assessed the anxiety levels experienced by clinical trainers, compared the prevalence of clinical anxiety among dental students and practitioners, and ranked typical clinical anxiety-provoking circumstances [15–17].

In this study, responses were collected from third and fourth year dental students regarding 34 clinical anxiety-precipitating situations. The results indicated an incidence of clinical anxiety of 60.8% of the whole participated dental students which was in accordance with previous studies reporting higher levels of clinical anxiety among dental students [15, 18, 19].

However, our study found no statistical significance regarding age related clinical anxiety among our 3rd and 4th year dental students which was in contradiction with previous study proposing that the level of clinical anxiety decreases progressively as students advance through their clinical training [19]. The reason for this finding could be explained by that dental students at the faculty of Dentistry, King Salman International University, were instructed and adequately prepared by the appropriate knowledge and classes prior to the beginning of their clinical work. However, in a few dental scenarios, our dental students either overstated or concealed their anxiety levels. This can be explained by the dental student’s ignorance or carelessness [20–22].

Moreover, female dental students in our study had higher anxiety score in all domains than males, which were statistically significant *P* < 0.001. This finding fit well with universally reported higher levels of anxiety among female students [23–25]This could be explained by the intrinsic psychological variance between genders in which females are more likely to articulate their worries and emotions [19].

Surprisingly, our study revealed higher anxiety score in all domains regarding dental students who reported dental phobia and previous bad dental experiences, which were statistically significant *P* < 0.016, *P* < 0.003 respectively. These findings could be clarified by Gazal et al. who suggested that those who struggle with anxiety disorders frequently deal with intrusive thoughts that don’t go away and may avoid particular situations out of fear [2].

It is commonly known that a number of dental school-related factors, including tough pre-clinical prerequisites, thorough exams, and the duty of providing patient care, can serve as strong inducers of clinical anxiety [26]. Additionally, the COVID-19 epidemic increased the complexity of dentistry education globally to a level never seen before [27]. A move to virtual learning platforms, limited access to clinical facilities, and interrupted patient care were among the particular difficulties brought on by the abrupt disruption of clinical training brought on by lockdown measures [28]. These interruptions have an impact on dental students’ clinical experience in terms of both number and quality.

Although earlier research provided insight into the frequency among dentistry students, our study went further by not only reporting the incidence of clinical anxiety among dental students but also looking at three domains “Clinical management requirements in general, Patient’s Diagnosis and management, Different dental treatment plan” including general and specific clinical anxiety related circumstances among these students.

Therefore, our study found 74.0 mean score for anxiety among the examined three domains including” different dental treatment plan procedures “domain related clinical precipitating situations as fulfilling the largest portion 31.2 out of 74.0. Accordingly, our dental students rated the clinical anxiety precipitating situations in a descending order as follows: extracting wrong tooth (39.5%), giving wrong treatment (37.6%), tearing of the cheek/lips due to catching on a dental burr (35.7%), fracturing a tooth (34.4%), wrong diagnosis (28.1%), inadvertently hurting the patient (22.1%), inability to meet requirements before exams (19.1%), dealing with a fainting patient during dental treatment (18.7%). Two clinical anxiety precipitating situations had almost the same score including restoration failure/recurrent caries and endodontic retreatment procedures 14.8% and 14.4% respectively. Also, extracting tooth and arresting postoperative bleeding had the same score of 13.7%, while the rest of other clinical situations ranged from 11.8 to 1.1%.

These results were consistent with, but somewhat different from, a prior study by Gazal et al. that found the most stressful dental situations were treating mentally ill patients, treating patients with medical impairments, dealing with uncooperative patients, dealing with fainting patients, fracturing a tooth, extracting the wrong tooth, exposing the pulp accidentally, fearing the patient’s satisfaction, tearing the cheek or lips after catching a dental burr, getting infected by patients, and administering the incorrect treatment [2].

Additionally, in agreement with Obarisiagbon et al., who discovered that the most common clinical anxiety-inducing circumstances were dealing with mental patients, coping with uncooperative patients, failing to meet prerequisites prior to exams, and failing final exams. Also, children, patients getting infected, a tooth breaking during extraction, removing the incorrect tooth, the supervisor finding calculus after scaling, unintentional pulp exposure, accidently injuring patients, and using the high speed hand piece [8].

Certain discrepancies in our study, such as the lower number of female students compared to male students, the fact that the majority of participating dental students lived outside of South Sinai, and the fact that our dental students either overstated or concealed their anxiety levels in a few dental scenarios, suggested that factors other than clinical exposure, like personal coping strategies and outside stressors, significantly influence how students experience anxiety [26].

## Conclusions

It was concluded that dental students in the study population had a comparatively high level of anxiety during clinical classes especially females and those with dental phobia and previous bad dental experiences. They rated the clinical anxiety precipitating situations in a descending order as follows: extracting wrong tooth, giving wrong treatment, tearing of the cheek/lips due to catching on a dental burr, fracturing a tooth, wrong diagnosis, inadvertently hurting the patient, inability to meet requirements before exams, dealing with a fainting patient during dental treatment. Two clinical anxiety precipitating situations had almost the same score including restoration failure/recurrent caries and endodontic retreatment procedures. Also, extracting tooth and arresting postoperative bleeding, while the rest of other clinical situations rated less than the others. Getting dental students ready to handle the stress brought on by taking care of the patients appears to be important. Dental students may benefit from stress management classes, mentorship programs, and improved clinical supervision in order to reduce anxiety and build resilience.

## Data Availability

Data is provided within the manuscript.
